# Clinical audit of multidisciplinary care at a medium-sized hospital in Spain

**DOI:** 10.1186/1477-7819-12-53

**Published:** 2014-03-06

**Authors:** Ana Ruiz-Casado, María Jesús Ortega, Ana Soria, Héctor Cebolla

**Affiliations:** 1 Medical Oncology Department, Hospital Universitario Puerta de Hierro-Majadahonda, C/ Joaquín Rodrigo 2, 28222, Majadahonda, Madrid, Spain; 2 Cancer Committee. Hospital Universitario de Fuenlabrada. Camino Molino 2, Fuenlabrada, Madrid, Spain; 3 Department of Social Stratification, Universidad Nacional Educación a Distancia, Calle Obispo Trejo, 2 28040 Madrid, Spain

**Keywords:** Multidisciplinary care, Cancer conference, Quality cancer care, Electronic clinical record

## Abstract

**Background:**

Multidisciplinary care is a key enabler in the provision of high quality care for cancer patients. Despite compelling evidence supporting their benefit to patients and for providers, multidisciplinary cancer conferences (MCC) are not universally occurring. Team composition of MCC reflects the multidisciplinary nature of the body. Lack of nursing input can have a negative impact on team decision making. The objective of this study was to evaluate multidisciplinary care and adherence to national recommendations at a medium-sized hospital through a clinical audit of cancer conferences and clinical records.

**Methods:**

A total of 77 multidisciplinary cancer conferences were visited and 496 electronic health records were reviewed. The regularity of meetings and multidisciplinary attendance were evaluated. Each electronic health record was checked to verify documented prospective discussion before any treatment was started.

**Results:**

Nine multidisciplinary teams meet on a weekly or biweekly basis at the hospital with an average number of ten people and six different specialties represented. Average duration of meetings was 46.8 min. Though most patients (64.5%) were discussed at some point at the relevant cancer conference, only 40% had a documented multidisciplinary team discussion prior to the first treatment. Pathological stage (pTNM) was documented in 53.6% of clinical records.

**Conclusions:**

Nursing representatives should be included as usual attendees at cancer conferences. Prospective discussion of all cancer cases should be encouraged. Use of checklists and systematic collection of key information, specifically cancer staging, could improve clinical documentation in the electronic clinical record.

## Background

Responding to growing concerns regarding safety, quality and efficacy of cancer care in the United States, the Institute of Medicine (IOM) commissioned a comprehensive review in the late 1990s [1]. In its follow-up report in 2000 the IOM recommended among other things: identification of a core set of evidence-based quality measures, standardization of reporting with regard to disease stage and reporting performance data. The IOM advocated for the enhancement of cancer care data systems through those mechanisms [2]. These reports have inspired several further national cancer plans. In Spain, a first national cancer plan was launched in 2006 [3] and was reviewed in 2009 [4]. Prospective evaluation of every cancer patient by a multidisciplinary team is recommended in both documents.

The quality of cancer care can be precisely defined and accurately measured. But there are many different perspectives to consider. Structural characteristics include clinician (board certification, distribution of specialties, *etcetera*) and organizational characteristics (staffing patterns, schedules, *etcetera*). Structural characteristics are necessary to provide good care but are insufficient to ensure excellent quality. Research suggests that outcomes are improved when patients receive cancer care from a highly functioning multidisciplinary care team (another structural characteristic). Multidisciplinary cancer conferences are not universally occurring, despite compelling evidence supporting their benefit to patients and for providers [5]. The evidence shows that the roles played by different team members within the multidisciplinary team (MDT) are varied, with lower importance placed on the input of nursing personnel [6]. Regarding multidisciplinary care, a high degree of variability could be expected in Spain in the context of a decentralized health care system [7]. In Spain there is not any kind of accreditation for hospitals providing cancer care, though in Catalonia, specialized cancer surgery has recently been limited to high-volume hospitals.

Monitoring MDT meetings activity ensures that conferences provide consultative services for patients to formulate an effective treatment plan and offer education to physicians and allied health professionals in attendance. The Hospital Universitario de Fuenlabrada (HUF)-Cancer Program requires routine evaluation of cancer conference activity in each of these five areas: 1) conference frequency, 2) multidisciplinary attendance, 3) total case presentation, 4) prospective case presentation, and 5) cancer staging.

Measurement and reporting of quality of care is an essential part of the conceptual framework for quality improvement. Our objective was to evaluate the multidisciplinary care of cancer patients and adherence to the Spanish cancer plan recommendations (‘Estrategia en Cáncer del Sistema Nacional de Salud’) [3,4] at a medium-sized Spanish hospital.

## Methods

The HUF has been the general hospital in the southwest region of the Comunidad de Madrid since June 2004. It has 406 beds. A unique electronic medical record is used.

### Audit of multidisciplinary team meetings

The Cancer Committee appointed four oncology nurses by March 2011 in order to audit the cancer conferences. Nurses were not usual members of the teams, so they could act as external evaluators. As hospital workers involved in patient care, they were allowed to review the medical information discussed at the meetings. The Cancer Committee provided the evaluators with a calendar of the annual cancer conferences schedule and the venue for each meeting.

External evaluators are responsible for checking: 1) that the conference was held at the place and time as scheduled in the calendar, 2) the starting and ending time, 3) the number of attendees, 4) the specialty of the attendees, and 5) the number of cases discussed.

### Audit of clinical records

All the 2009 incident cases registered by March 2011 (hospital-based tumor registry) were scheduled to be reviewed in 2011 by the Cancer Committee members (in parallel with the process of the MDT meetings audit). The criteria to be reviewed had been previously agreed upon by the members of the Cancer Committee according to the Hospital-Cancer Program: 1) the recommendation made by the MDT was documented in the clinical record; 2) the discussion was held before any treatment had been done; 3) the TNM staging should be documented; 4) the diagnostic interval (date of diagnosis minus date of first contact); and 5) the therapeutic interval (date of first treatment minus date of diagnosis). The diagnostic and therapeutic intervals were calculated for review purposes. Date of first contact is the first time the patient contacts the hospital (that is, the date the patient asks for an appointment and not the date of the appointment). The date of diagnosis required not only pathologic confirmation (when possible) but also the necessary work-up for decision making.

## Results

### Audit of cancer conferences

Nine cancer conferences were identified by the Cancer Committee: breast, dermatology, gastrointestinal (G-I), gynecology, head and neck (H and N), hematology, lung, sarcomas and urology. Five conferences (breast, G-I, lung, hematology and urology) were planned on a weekly basis whereas the other four (dermatology, gynecology, H and N, and sarcomas) were planned every two weeks. Seventy-seven meetings were visited between April and October 2011. All the meetings were held at the planned location, starting with a median delay of 5.6 min (−5 - +15). Average duration of the meetings was 46.8 min (15 to 95). Average number of attendees was 10.4 (3 to 31). Average number of different represented specialties was 6.1 (3 to 11). There were no nurses among the regular attendees, although during the audit period nurses were present as external evaluators.

**Figure 1:**
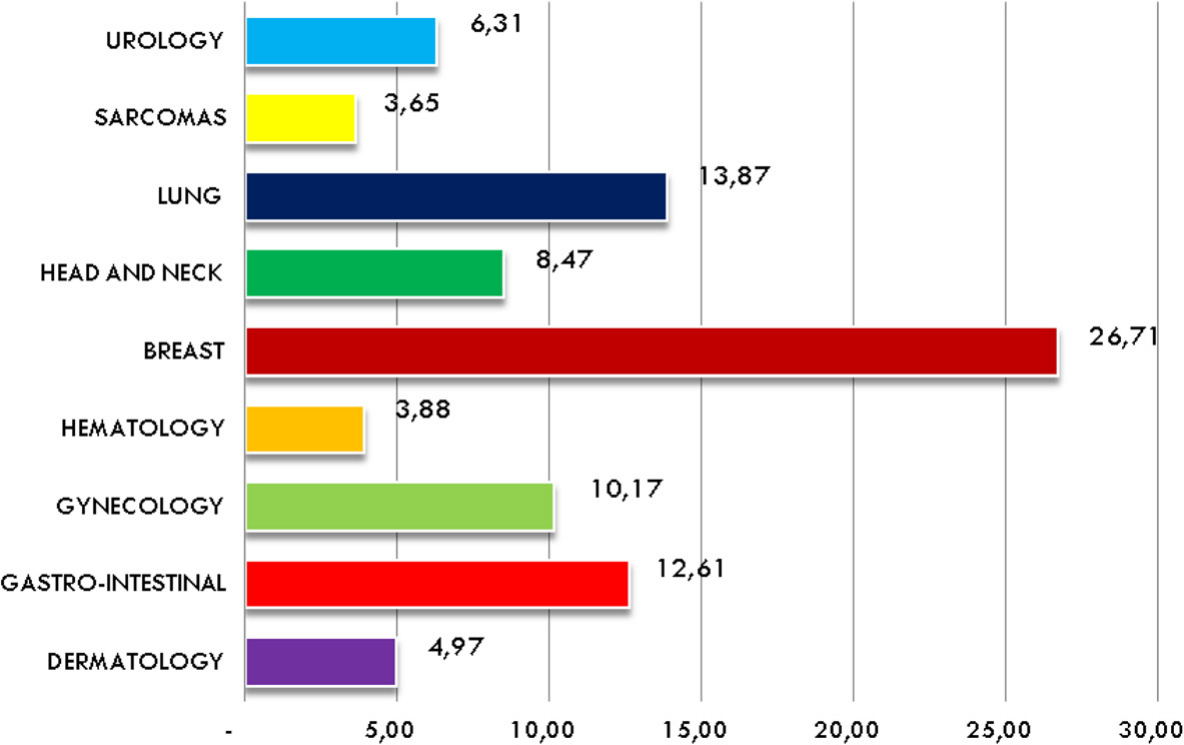
Cases per meeting.

A post-hoc analysis tried to identify if the so-called core members were present in all meetings. To define core members, we referred to ‘Multidisciplinary Cancer Care Tools’ [8]. The participants required to make a qualified decision (core-members) were: the medical oncologist, the radiation oncologist, the surgical oncologist, the pathologist and the radiologist. The hematology cancer conference did not require the attendance of a surgical oncologist and required the presence of a hematologist instead of a medical oncologist. All core members were present in 42 out of 77 visited meetings (54%).The number of cases discussed per meeting varied among the different cancer conferences and was higher for conferences reviewing high-incidence tumors such as breast, lung and G-I (see Figure 1). The average cases discussed per meeting was 7.8. The average duration of discussion per case was 6 minutes. For this analysis it should be clarified that case does not necessarily mean patient (some patients were discussed several times). A specific retrospective analysis was done to distinguish between cases and actual patients presented at the gastrointestinal and breast cancer conference. The 526 cases in the breast cancer conference were for 129 patients, whereas the 162 cases in the gastrointestinal cancer conference were for 149 patients.

Table 1 shows the data per cancer conference.

**Table 1:** Audit of cancer conferences

**Cancer conference**	**Visited meetings**	**Punctuality**	**Duration**^**a**^	**Attendees**^**a**^	**Specialties represented**	**Qualified quorum**	**Cases**	**Time (min) per case+**	**Patients**^**b**^
Breast	19	+10	70 (40 to 95)	10 (4 to 13)	7 (4 to 10)	13	526	3 (2 to 4)	129
Dermatology	3	+5	28 (15 to 40)	7 (6 to 7)	6 (5 to 7)	2	15	6 (3 to 7)	-
Gastrointestinal	12	−5	51 (40 to 75)	26 (7 to 31)	8 (5 to 11)	10	162	4 (2 to 7)	149
Gynecology	5	+5	46 (35 to 75)	7 (5 to 8)	5 (3 to 6)	2	53	4 (3 to 6)	-
Head and neck	7	+5	49 (35 to 65)	10 (10 to 11)	7 (6 to 8)	7	64	5 (3 to 10)	-
Hematology	11	+15	37 (12 to 75)	8 (5 to 11)	5 (4 to 6)	4	47	9 (6 to 13)	-
Lung	12	+6	46 (30 to 75)	8 (3 to 11)	5 (3 to 7)	0	173	3 (2 to 5)	-
Urology	3	0	48 (30 to 75)	14 (13 to 15)	7 (6 to 8)	1	19	7 (4 to 12)	-
Sarcomas	5	+10	51 (40 to 75)	6 (4 to 9)	5 (4 to 8)	3	21	13 (8 to 18)	-
Total	77	+5.6 min	46.8 min	10.4	6.1	42	1080	6 min	-

^a^Average (range).

^b^This analysis could not be done for every cancer conference.

### Audit of clinical records

A total of 496 cases were generated by the hospital-based tumor registry. Ten members of the Cancer Committee reviewed the e-health records. A total of 320 (64.5%) patients were discussed in different cancer conferences (according to the minutes of the meetings). A total of 259 (52%) patients had written documentation of the MDT recommendation in the clinical record. A total of 198 (40%) patients had had an MDT discussion prior to the first treatment.

Only TNM staging was considered. Clinical staging (cTNM) was recorded in 118 (23.8%) and pTNM in 266 (53.6%) of the clinical records.

The median diagnostic delay was 33 days and the median therapeutic delay was 21 days.

Table 2 shows the clinical record data per cancer conference.

**Table 2:** Audit of clinical records

**Relevant cancer conference**	**Cases**	**MDT discussion**	**Prospective presentation**	**Written documentation**	**Explicitly documented cTNM**	**Explicitly documented pTNM**	**Diagnostic delay**	**Therapeutic delay**
General	496	320 (64.5%)	198 (40%)	259 (52%)	118 (23.8%)	266 (53.6%)	33	21
Gastrointestinal	147	124 (84.3%)	87 (59.1%)	116 (78.9%)	58 (39.4%)	89 (60.5%)	30	21
Breast	46	43 (93%)	38 (82.6%)	43 (93%)	9 (19.5%)	35 (76%)	28	28
Lung	68	36 (52.9%)	15 (21.7%)	10 (14.5%)	11 (16.1%)	24 (35.3%)	13	10
Hematology	33	14 (42%)	7 (21%)	8 (24%)	na	na	na	na
Urology	81	46 (56.7%)	15 (18.5%)	28 (34.5%)	11 (13.5%)	52 (64.2%)	46	33
Gynecology	23	19 (82.6%)	10 (43.5%)	16 (69.6%)	5 (21.7%)	16 (69.6%)	34	31
H and N	28	16 (57%)	8 (28.6%)	8 (28.6%)	11 (39.3%)	19 (67.8%)	46	19
Sarcomas	5	5 (100%)	2 (40%)	1 (20%)	1 (20%)	3 (60%)	41	15
Melanoma	16	14 (87.5%)	2 (40%)	11 (68.7%)	3 (18.7%)	11 (68.7%)	13	56
Other: Unknown origin, Thyroid, Brain, Peritoneum	41	4 (9.7%)	2 (4.9%)	1 (2.4%)	1 (2.4%)	7 (17.1%)	50	23

H and N, head and neck; na, not available due to the specific characteristics of hematological cancers. MDT (multidisciplinary team); cTNM (clinical staging); pTNM (pathological staging).

## Discussion

Cancer care can be complex. Due to the large number and range of healthcare providers who may be involved, there is potential for poor communication and poor coordination of care. Multidisciplinary care (MDC) has been identified as a key enabler in the provision of high-quality treatment and care for cancer patients [9].

The evidence for improved survival as an impact of multidisciplinary cancer care is not definitive. A Scottish study linked substantially greater improvements in breast cancer survival to MDC [10]. A Norwegian study also showed a twofold increase in survival of upper gastrointestinal patients [11]. However an American study in the Veterans Affairs health system provides some evidence against that previous statement [12]. These studies did not have the strength of randomized controlled trials, which are no longer possible because of the prolific introduction of the multidisciplinary approach. Nonetheless, MDC is purported in the literature to offer many benefits. It reduces time to diagnosis and treatment, improves adherence to guidelines, improves inclusion in clinical trials, improves patient satisfaction and improves education and collegiality for members of the MDT [9]. Cancer conferences are used for the attainment of accreditation [13].

**Figure 2:**
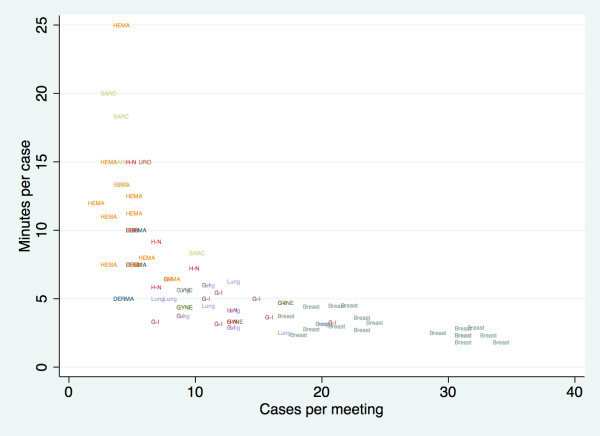
Time per case and cases per meeting.

To be considered as an additional structural characteristic of the hospital, every meeting should be periodic and fixed at a preplanned location. MDT members should have dedicated time included in their job plans to prepare and attend MDT meetings. These meetings should be held during core hours and should not clash with related clinics that members need to attend [14]. Organizational factors related to the structure of the MDT meeting are associated with variation in the likelihood of reaching a treatment decision. A recent study revealed that an increased number of cases per meeting and team members in attendance, as well as more time per case, were associated with better teamwork [15]. Our results could help other organizations to estimate the protected time for this purpose. Regarding time per case, we obtained similar figures to the published ones for GI meetings (around 4 minutes in the United Kingdom) [16], but quite longer for urological meetings (around 2 minutes in the UK) [14]. Perhaps it is not surprising that the longest time devoted per case was for low incidence and complex tumors such as sarcomas (Figure 2). One of the well-known advantages of cancer conferences is the establishment of clinical management protocols and development of treatment pathways [17]. This approach works better in high-incidence tumors such as lung, breast or colorectal cancer.

The IOM report, the National Institutes of Health (NIH) guidance as well as the ‘Estrategia en Cáncer’ recommend that every cancer patient should be discussed at an MDT before a treatment is started. However, many professionals argue that this approach is not feasible because of time and economic restrictions [18]. Though most cancer cases in this audit were discussed at some point by an MDT, fewer than half of the overall population were prospectively reviewed.

Multidisciplinary composition is based on the presence of different specialties in the meeting. An effective multidisciplinarity should include real participation and not only physical presence. But evaluation of this functional aspect was beyond the scope of our evaluation. Our Hospital-Cancer Program did not establish whether the presence of some specialists was specifically required. We performed a post-hoc analysis in order to understand whether we would be compliant for a qualified quorum [8]. One of the findings was that because thoracic surgery was not an in-house discipline, a qualified quorum could not be reached in any of the lung cancer conferences. But even including that conference, 54% of the audited meetings were attended by the tumor-specific minimum core team, much more than the 1% obtained in a recent survey in Australia [19] and similar to the 49.3% observed in The Netherlands [20].

Nurse navigators are a critical component to the success of an MDT. Their role should be to coordinate and develop a plan of care with physicians, coordinate appointments, disseminate information and provide information and education to patients and family members. It has been demonstrated that a bias towards biomedical information occurred when nurses do not participate [21]. Nurses make a valuable contribution to clinical decision making in the MDT meeting with particular focus on patients’ comorbid health, psychosocial and social issues [22]. Dominance of the medical profession in the healthcare system is one of the main barriers to this kind of collaborative practice [23].

The medical record is an essential source of information on the delivery of care as well as a measure of proficiency [24]. The use of an electronic clinical record (ECR) has potential to decrease the amount of missing clinical information but cannot solve all the problems. In fact, we found some difficulties in locating some specific information because there was no structured procedure for collecting information in the ECR. Essential information should be codified, easily found and ready for semantic interoperability. A poor medical recording performance is related to a poor medical care performance [25], and poor physical documentation could falsely suggest that rates are worse than they really are [26]. The lack of key information in the ECR, such as performance status (PS) assessment or TNM, staging could be overcome through checklists and pro-forma practices. A locally agreed minimum dataset of information about patients to be discussed should be collated and summarized prior to MDT meetings and it should be in line with data items that are in existing national datasets. We propose the use of a checklist for MDT discussion since it has been considered that the recommendations are only as good as the information on which they are based.

Cancer staging is a process that describes the anatomic extent of a tumor. It is also a prognostic variable and allows comparability. It is universally accepted that staging is an essential component of cancer management. It should be considered before deciding a treatment, and it should be explicitly documented. Documentation of clinical and pathological staging is considered a quality indicator also linked to the clinical record quality. Whereas clinical staging should always be possible, pathological staging requires a previous resection. On the other hand, TNM staging is not accepted for all cancers (for example, hematological cancers are not TNM staged). For our analysis, standards would not be 100% because of these considerations. But we still think that clinical staging documentation was clearly insufficient in our audit. A lack of appropriate skill or motivation is the most frequent cause for an inaccurate staging [27]. The National Cancer Institute of Canada adopted a national policy in the 1990s to consider staging a standard of care [28]. Motivation program have demonstrated usefulness in this setting [29].

In our audit, more than half of the cases (64.5%) were reviewed by the relevant MDT, with the decision documented in the clinical record in 52% of cases. These figures compare favorably with the ones obtained in 2007 by this Cancer Committee, when 41% of the cases had been discussed. Only 40% of the cases overall were prospectively discussed. This was the main finding of this audit, with improvement clearly being necessary for our standards.

Diagnostic and therapeutic delays are not comparable because of our particular definition of date of diagnosis requiring complete work-up. These figures will be more useful for proposing some in-house improvements than for use in comparisons with other settings. In any case, they also compare favorably with other published data [30].

We have to acknowledge some weaknesses in our evaluation. We reviewed the 2009 registered cases by March 2011, but additional cases were subsequently registered (up to 869) because of the retrospective nature of cancer registration. In this regard we recognize a selection bias. We analyzed some quality criteria related to MDC, but some others, such as documentation of familial antecedents or prognostic indicators (that is, PS, preoperative carcinoembryonic antigen, *etcetera*) in the clinical record were not analyzed. And probably, the main weakness of this study was the lack of information about the decision-making process and the degree of implementation of the recommendations made by the MDT. This aspect would have required a different methodological approach.

## Conclusions

To sum up, we can conclude that nine site-specific, multidisciplinary teams meet regularly on a weekly or biweekly basis at the Hospital Universitario de Fuenlabrada. The absence of nursing representatives was the main finding regarding team composition. Most cases of cancer were reviewed by an MDT, but only 40% were discussed before any treatment was initiated. Clinical documentation improvement is necessary. We propose the use of checklists for MDT discussion. Electronic health records require organizations to establish standards for functionality, data representation and interoperability. Key information should be collected in predefined locations.

Clinical audits are seen as one approach to improving the quality of patient care. Sharing knowledge and expertise on different models for comprehensive and integrated cancer care and, in particular, organization of care, are actions recommended by Commission of the European Communities [31]. A coordinated team-based cancer care is still advocated by the latest relevant IOM report [32], 15 years after the publication of the transformative ‘Ensuring Quality Cancer Care’ [1].

## Abbreviations

cTNM: Clinical staging; ECR: Electronic Clinical Record; GI: Gastrointestinal; IOM: Institute of Medicine; MCC: Multidisciplinary Cancer Conference; MDC: Multidisciplinary Care; MDT: Multidisciplinary Team; NIH: National Institutes of Health; PS: Performance Status; pTNM: Pathological Staging; TNM: staging system developed and maintained by the UICC.

## Competing interests

The authors declare that they have no competing interests.

## Authors’ contributions

ARC contributed substantially to the conception and design of the study. She analyzed the data from health records audit. MJO and AS visited the cancer conferences. They contributed substantially to the acquisition of data from cancer conferences audit. They analyze the data from cancer conferences audit. ARC, MJO, AS and HC contributed substantially to the whole analysis and interpretation of data. All authors read and approved the final manuscript.
